# Alcohol consumption and closed borders - how COVID-19 restrictions have impacted alcohol sales and consumption in Europe

**DOI:** 10.1186/s12889-022-13014-1

**Published:** 2022-04-08

**Authors:** Håkan Leifman, Kalle Dramstad, Emil Juslin

**Affiliations:** 1 Department of Clinical Neuroscience, Karolinska Institutet, Stockholm, Sweden; 2 IOGT-NTO, Stockholm, Sweden

**Keywords:** COVID-19 pandemic, Cross-border alcohol trade, Alcohol sales, Substitution, Alcohol consumption, Crisis-response impact

## Abstract

**Background:**

The closing of bars, restaurants and international borders during the COVID-19 pandemic led to significant changes in alcohol availability. This study provides a first systematic overview of the monthly development of alcohol sales in Europe during the pandemic in order to determine the effect of closed borders on the sales and consumption of alcohol.

**Methods:**

The study covers 72 months from January 2015 to December 2020 in 14 countries from northern, central and western Europe with excise revenue data for beer, spirits, wine separately and summed, converted into litres of pure alcohol per capita 15+ as a proxy for alcohol sales. March–December 2020 is seen as the pandemic period. The analyses consist of (1) descriptive trends of sales before and during the pandemic, (2) assessment of the pandemic impact on sales by time-series analyses and (3) case studies of countries and a region with substantial cross-border inflow or outflow of alcohol.

**Results:**

The result shows an overall reduction in alcohol sales with 3.6% during the pandemic. Nevertheless, the results differ based on the level of cross-border purchasing flows pre-pandemic, as countries with high cross-border inflow saw an increase in domestic sales as the pandemic hit. Norway, for example, saw a 23% increase in domestic sales during the pandemic period March–December 2020 compared to the same period in 2019.

**Conclusion:**

The closing of intra-European borders had a significant redistributing effect on alcohol sales. While noting sales increases, cross-border inflow countries generally saw a decrease in total amount of alcohol consumed per capita as not all cross-border purchases were replaced by domestic sales. This has important policy implications as large volumes of cross-border inflow of alcohol can negatively affect excise revenue as well as public health outcomes.

The methodology can be used to further explore the reliance of different purchasing streams in a domestic alcohol market.

**Supplementary Information:**

The online version contains supplementary material available at 10.1186/s12889-022-13014-1.

## Introduction

The COVID-19 pandemic has had a profound effect on individuals and communities throughout the world, including in Europe. As the pandemic required rapid reorganisation of societies, it has given rise to unique research opportunities, not the least in the alcohol policy field.

Already in May 2020, Rehm et al. predicted that two mechanisms would affect alcohol consumption during the Covid-19-pandemic [1]. The first mechanism, hereafter referred to as crisis impact, relates to the structural effects inherent to the pandemic itself. It is caused by increased psychosocial distress triggered by the interaction of financial difficulties, job losses and uncertainty about the future. This was predicted to lead to an increase in alcohol consumption and more harmful consumption patterns. The crisis impact mechanism has been apparent among citizens all over Europe, but the degree of psychosocial stress seems to have varied between different European countries [2].

The second mechanism, hereafter referred to as crisis-response impact, relates to the effect on alcohol consumption of the measures enacted in response to the pandemic. While some of the crisis-response measures led to similar effects as the first mechanism, such as psychosocial distress, the mechanism in itself also had other effects. In Europe, most countries, if not all, took  actions aiming at restricting physical and social contact and several of these measures directly and indirectly targeted alcohol consumption. This included restrictions on alcohol sales at restaurants, pubs and bars as well as bans on social gatherings. Therefore, the crisis-response measures were predicted to reduce alcohol consumption.

The crisis-response impact also exhibits similarities across countries. As shown by the Oxford COVID-19 Government Response Tracker (OxCGRT), the overall stringency of these measures is rather similar across European countries, although with somewhat different actions taken and different dates for its implementation [3].

This study, however, focuses on an asymmetrical impact of the crisis-response on alcohol consumption across countries, namely the effects of closed borders and severe restrictions on cross-border travel on sales and consumption of alcohol.

### Alcohol consumption and closed borders

The possibility of travel abroad fell sharply during the pandemic (see [3, 4]) and thereby restricted the opportunity for residents in countries with relatively high alcohol prices to purchase alcohol in lower-price countries to bring back home. As such, border restrictions had an asymmetrical impact on alcohol availability by affecting residents in European countries with a large share of cross-border alcohol purchase inflow (inflow countries) differently to residents in countries with low net levels of cross-border alcohol movements (stable countries) or countries with a large share of cross-border alcohol purchase outflow (outflow countries).

In line with previous alcohol policy research, restrictions in alcohol availability, whether it is affecting on-trade, off-trade or cross-border purchases, would be expected to reduce alcohol consumption even when taking into account some reallocation between or within purchasing channels (see e.g. [5]).

For inflow countries, a large share of alcohol consumed by its residents is not recorded within the country and for outflow countries, recorded sales include a large share of alcohol that is purchased by visitors and consumed outside the country. In these countries, recorded alcohol consumption (domestic sales - hereafter also referred to as RAC) thus diverges from actual consumption. Estimations of the actual level of per capita alcohol consumption is therefore a challenge (for an overview of unrecorded alcohol in Europe, (see [6, 7]).

The closing of borders during the pandemic, however, meant that RAC also in these inflow and outflow countries can be expected to closely reflect actual domestic alcohol consumption, providing a unique possibility to study this phenomenon in a comparative context.

In Europe, there is a historical concentration of cross-border shopping to two primary border regions: Nordic-Baltic and Benelux-France + the British Isles (see [8, 9]). Monitoring the trends in RAC in these countries should thereby provide insights into the impact of the pandemic on RAC in a cross-border context. Comparative analysis of the asymmetrical effect of cross-border restrictions between countries should in turn improve our understanding of the magnitude and movements of alcohol between different countries in Europe.

### Cross-border purchases of alcohol in Europe

In a recent overview study on the cross-border trade of alcoholic beverages within the European Union (EU), three main concerns associated with cross-border alcohol purchases were identified: its impact on public health, on fraudulent behaviour (e.g. smuggling) and on economic distortion [9]. All three share the same underlying causal mechanisms driven by (a) alcoholic beverages being price sensitive (e.g. [10, 11]) (b) the existence of substantial excise duty differences (and consequently price differences) between EU countries (see e.g. [12]) (c) the legal possibility in the EU to cross from one EU country to another with almost unlimited quantities of alcohol for own use without further payment of excise duties in the country of consumption.^1^

From a public health point of view, extensive cross-border inflow of alcohol may increase total per capita alcohol consumption,^2^ and as a consequence, alcohol-related problems, through two main processes. Firstly, by directly increasing accessibility of cheaper alcohol. Even if residents substitute some domestic purchases with purchases abroad, the net effect of cross border purchases is likely an increase in total alcohol consumption as an effect of access to alcohol at a lower price. Secondly, by dampening the will to use excise duties as a public health tool for fear of losing purchases to neighbouring states. As an outspoken reaction to the significant inflow of alcohol, Denmark, Estonia, Finland and Sweden have for the last decades taken decisions to lower the excise rates, either actively by tax cuts (e.g. Sweden in 1997; Denmark in 2003 and 2019; Finland in 2004 and Estonia in 2019) and/or by keeping the rates on a stable nominal level.

Data and most, but not all, studies from these inflow countries in northern Europe have provided evidence of both of these two mechanisms (Denmark: [16]; Estonia: [17, 18]; Finland: [19, 20]; Sweden: [21, 22]). Studies in other countries in Europe are only fragmentally available. A recent survey study of 6250 respondents across 25 EU Member States in 2018/2019, however, confirms the pattern of significant variations in cross-border alcohol purchasing across countries, with high priced countries tending to show the highest volumes [9].

Table 1 provides an overview by categorising countries as inflow, outflow or net stable depending on knowledge of the cross-border purchase streams before the pandemic. Where possible, information is taken from national alcohol consumption surveys. Where such surveys do not exist, a triangulation of excise duty rate differences, the presence of specialised border shops and news articles are used (see Additional file 1: Table S1).

**Table 1:** Preliminary categorisation of countries in relation to cross-border purchase flows

Study countries	Category
Belgium (BE)	Net stable/inflow
Germany (DE)	Net stable / outflow
Denmark (DK)	Inflow
Estonia (EE)	Inflow/outflow
Finland (FI)	Inflow
France (FR)	Net stable / outflow
Ireland (IE)	Net stable / Inflow
Lithuania (LT)	Net stable / inflow
Latvia (LV)	Outflow
Luxembourg (LU)	Outflow
The Netherlands (NL)	Net stable
Norway (NO)	Inflow
Poland (PL)	Net stable
Sweden (SE)	Inflow

* See Additional file 1: Table S1 for data and comments

Four typical inflow countries are identified with a net balance of substantially higher volumes of inflow than outflow (SE, NO, FI, DK). Two countries are identified as typical outflow countries (LV and LU). A few countries are identified as having higher outflow than inflow or vice versa for some beverages but low or net stable flow for others. Remaining countries are classified as net stable.

### Hypothesis

The restrictions put in place during the Covid-19 pandemic to limit the spread of infection represent an unprecedented reduction of the physical and economic availability of alcohol for all residents in the affected territories. This applies to Europe in general, and inflow countries in particular. Following the prediction of Rehm et al. [1] one would therefore expect a reduction in alcohol consumption from the start of the pandemic in all, or a majority of European countries. Results from recent analyses of survey data on drinking frequency also suggest that this has been the case [23]. However, the lack of a clear connection between frequency and volume in self-reported data and alcohol consumption in surveys usually being substantially underestimated [24] makes it difficult to draw definitive conclusions with regard to the effects on total per capita alcohol consumption. This paper will test the hypothesis that total alcohol consumption has fallen during the pandemic in the European countries included in the study.

The crisis-response impact on cross-border alcohol purchases is of particular interest since trends in RAC is affected by changes in volumes of cross-border purchases. Operationalising the hypothesis and taking into account the effect on RAC of changes to cross-border purchases, this paper predicts the pandemic to have the following effect on RAC:

- **H1**: RAC will show a decreasing trend from the pandemic in countries with low levels, or net low levels, of cross-border purchases of alcohol.
- **H2:** RAC will show an increasing trend from the pandemic in cross-border inflow countries compared to H1 countries.
- **H3:** RAC will show a stronger decreasing trend from the pandemic in cross-border outflow countries compared to H1 countries.

Following from the hypothesised effects on RAC, one could expect H1 results to be indicative of effects on total consumption.

Previous studies on the relaxation of cross-border purchasing rules have found a degree of additionality to cross-border alcohol purchases, meaning they are not just replacing domestic sales, but contributing to an increase in total alcohol consumption. Consequently, inflow countries in H2 should see a stronger decreasing trend in total consumption than the other countries in the study, despite an expected RAC increase, as cross-border alcohol accessibility is reduced. In addition, the change in total alcohol consumption in outflow countries should be smaller than decreases in RAC, likely similar to H1 countries.

Furthermore, looking at RAC development across different categories of countries and combining it with findings from individual country studies on cross-border purchases, it should also be possible to provide a rough estimate of any potential degree of additionality in purchases stemming from access to cheaper alcohol across a border. Although going beyond the testing of the  three main hypotheses, looking at a potential additionality is of interest as previous studies have only ever been able to look at situations of increased accessibility of cross-border alcohol.

## Method

### Data

The study uses time series data on monthly alcohol excise duty receipts (tax records 2015–2020). Excise duty receipts represent all legal sales in a given territory, both off- and on-premises. This data is divided into separate beverage categories of beer, spirits and wine. The total RAC is a summary of all beverage categories and also includes intermediate products and other less common beverage categories, where available.

Data has been collected for 14 countries in northern, central and western Europe. There are minor differences in the data: for the Netherlands, quarterly income data is used and for Ireland data from 2016 to 2020 is used. (See Additional file 1: Table S2 for country information on data used). For Luxembourg, data is only available for spirits and only until October 2020, therefore Luxembourg is analysed separately from the other countries.

### Adjustments and conversions

By using historical excise duty rates, the data on excise duty revenues have been converted to volumes sold in 100% alcohol and divided by population aged 15 or over. For most countries, excise duties for beer and ethyl alcohol (below referred to as spirits) is based on alcoholic strength [12] and excise revenues can thereby be converted to 100% alcohol through historical excise duty rates. All countries in this study, except Germany and Luxembourg, levy excise duty on wine. As most excise duties on wine are structured by tax-bands and levied by litre finished product instead of alcohol content, the conversion into 100% alcohol is based on the most common tax band, usually covering wines between 8 and 15%.

### Measures

The outcome measures are the estimated level of total and beverage specific (beer, spirits, wine) monthly RAC in litres of 100% alcohol per capita age 15 or older^3^ 2015–2020. The independent intervention indicator is the months of the pandemic (March–December 2020) compared to the pre-pandemic months (Jan 2015-Feb 2020).

Excise duty rates were also used in order to control for the potential confounding effect of changes in excise rates on the pandemic impact estimates in Estonia, Latvia, Lithuania and Poland, since these countries had recent, more-than marginal, changes in excise rates. The data shows the rates per litre alcohol (100% for beer and spirits; volume litres for wine) in the local currency. In analyses of the impact on the total RAC, an overall taxation level measure was constructed based on a weighted sum of beer, spirits and wine taxation levels in relation to their contribution to the total RAC.

### Analysis

The analysis consists of three parts. Firstly, the monthly data is grouped into three time periods for every year: January–February, March–December and January–December. Descriptive trends in RAC for these three periods are presented for each country and for all countries taken together. March–December 2020 is considered as the pandemic period and is compared to the same periods for previous years. January–February for all years, including 2020, should be seen as an additional pre-pandemic control variable. The January–December measure is included since annual figures facilitate comparisons with other relevant data that are often reported per calendar year.

Secondly, a country specific pandemic impact assessment was carried out using an interrupted time–series (ITS) analysis on both total and beverage specific RAC. The time-series analysis was performed using a seasonal extension of the ARIMA modelling technique developed by Box & Jenkins [25], called SARIMA (Seasonal Autoregressive Integrated Moving Average). The specification of the SARIMA-models is done individually for each country and beverage, but all models are based on seasonally differenced data (see also Additional file 1: Table S3).

The following models were used for each country:

$$\mathit{\nabla}{LnA}_t=a+{\beta}_1\mathit{\nabla}{I}_t\left(+{\beta}_2\mathit{\nabla}{LnT}_t\right)+\mathit{\nabla}N$$

A signifies the RAC (total and beverage specific). I is the intervention variable (coded 1 for the pandemic months March–December 2020 and 0 all other months) and, when included, T is the taxation level. The taxation level T is only included in the model for countries with recent changes in taxation (2019 or 2020) and only included in final models where significant (most countries had no or only marginal changes in excise duty rates 2015–2020). The noise term N includes other etiological factors. The operator ∇ means that the series is differenced. Both the output (A) series and the input (T) series were logged and the intervention coded 1 or 0. The parameters for the intervention (β_1_) show the change in RAC during the pandemic period (the change in level from the pre-intervention to the intervention period).

The third part of the analysis consists of detailed case studies of the outflow region Schleswig-Holstein, a federated state in northern Germany, and the inflow country Sweden. The former is done in order to determine the impact that this region may have on the overall estimates for Germany and the latter is done in order to study the level of reallocation and potential additionality between cross-border purchases and domestic sales.

## Results

### Assessment of RAC trends before and during the pandemic

**Fig. 1:**
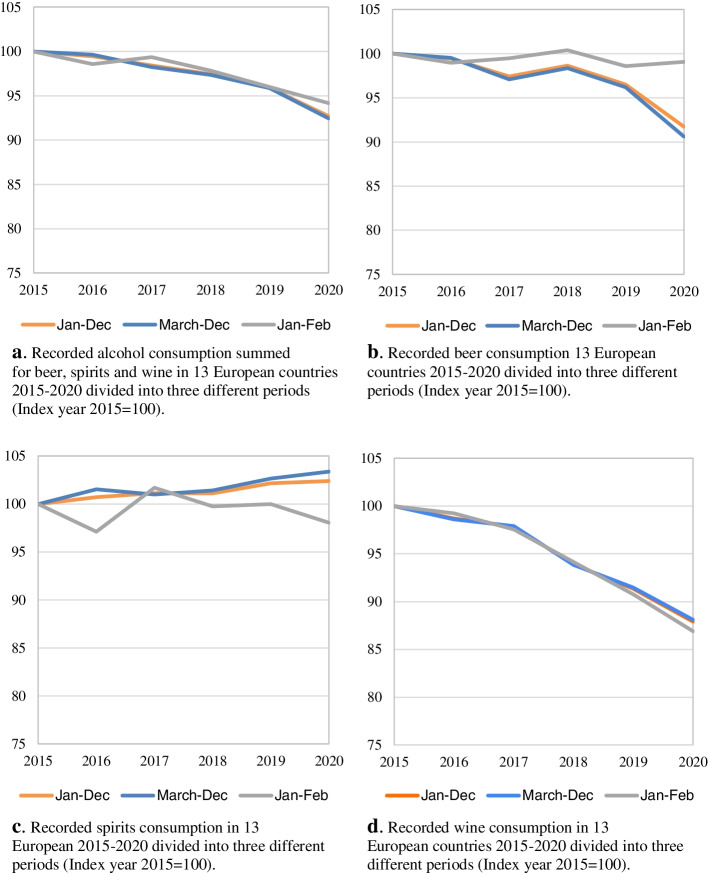
**a** Recorded alcohol consumption summed for beer, spirits and wine in 13 European countries 2015–2020 divided into three different periods (Index year 2015 = 100). (LU not included in **a**-**d** due to non-complete annual time series data (see methods section)). **b** Recorded beer consumption 13 European countries 2015–2020 divided into three different periods (Index year 2015 = 100). **c**. Recorded spirits consumption in 13 European 2015–2020 divided into three different periods (Index year 2015 = 100). **d**. Recorded wine consumption in 13 European countries 2015–2020 divided into three different periods (Index year 2015 = 100)

For all countries taken together (Fig. 1a), the total volumes of RAC (indexed, 2015 = 100) decreased by approximately 3.6% (− 0.31 l), during March–December 2020 compared to the same period 2019, and a 3.3% decrease for the calendar year. The slope of the decline 2019–2020 is stronger than for previous years. Furthermore, January–February 2020 shows rather stable total RAC levels compared to 2019 and does not follow the same trend as the ensuing pandemic period.

As shown in Fig. 1b-d, only beer shows a stronger downward trend during the pandemic months compared to pre-pandemic months in 2020 and 2019. From March to December 2020, the level of beer RAC decreased by 5.8% (− 0.21 l) compared to the same months in 2019. The volumes of wine also decreased during the pandemic months (− 3.7%, − 0.11 l) but the slope of the decline does not deviate from previous years. Spirits show a stable level during the pandemic period (+ 0.7%, + 0.01 l compared to 2019), also compared to previous years. For January–February, however, spirits RAC decreased by 1.9% in 2020, but similar changes can be observed also in previous years.

**Fig. 2:**
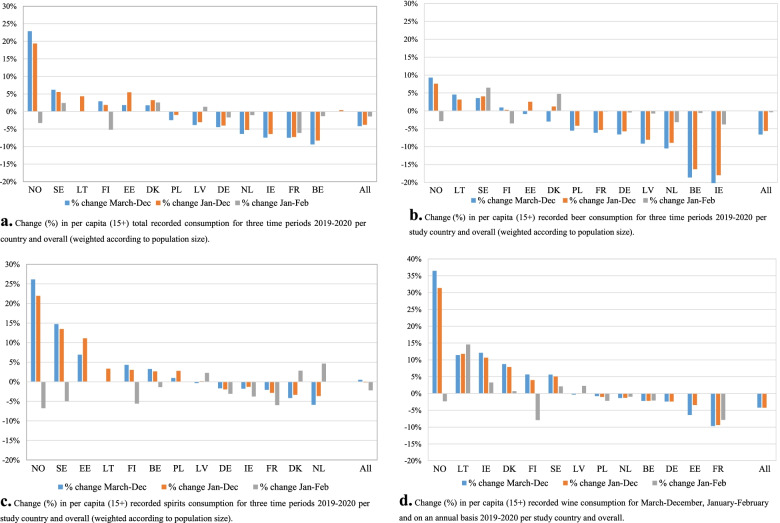
**a** Change (%) in per capita (15+) total recorded consumption for three time periods 2019–2020 per country and overall (weighted according to population size). (January–February data for EE (**a-d**), LT (**a-c**) and PL (**a-c**) and March–December for LT (**a, c**) not shown due to spikes related to taxation changes. For DK, data for December and January cannot be separated, therefore the data refers to February–March (**a-d**). LU not included in **a-d** due to non-complete annual time series data (see methods section)). **b.** Change (%) in per capita (15+) recorded beer consumption for three time periods 2019–2020 per study country and overall (weighted according to population size)^1^. **c.** Change (%) in per capita (15+) recorded spirits consumption for three time periods 2019–2020 per study country and overall (weighted according to population size). ^1^. **d.** Change (%) in per capita (15+) recorded wine consumption for March–December, January–February and on an annual basis 2019–2020 per study country and overall^1^

As shown in Fig. 2a, the aggregate decline is not synonymous with a decline in RAC across all countries, with six countries experiencing an increase and the others a decrease in RAC. Fig. 2b-d show the changes for each of the beverages for the same three periods. Beer shows not only the largest overall decline during the pandemic periodbut also more countries show a decline in beer compared to spirits and wine. Furthermore, for all the countries with an increase in total RAC (NO, SE, LT, FI, EE, DK), it is predominantly spirits and/or wine that contribute to the increase in the total RAC level. For these countries, beer shows a more modest increase or even a small decrease. Moreover, for most countries, the change in January–February 2020 is modest and does not point in the same direction or the same degree of change as the March–December period 2020.

**Fig. 3:**
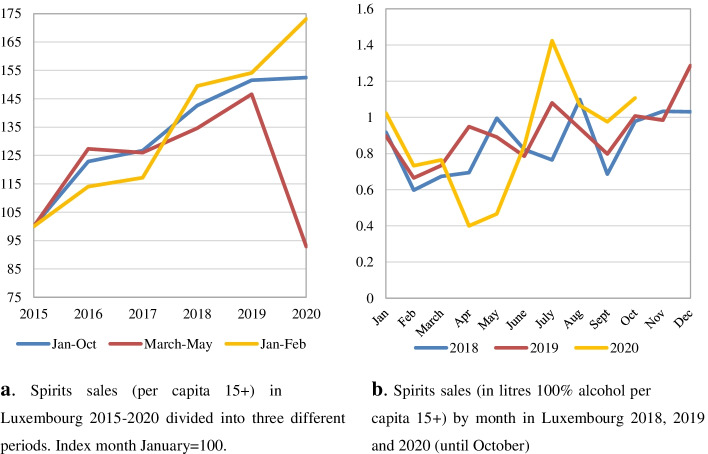
**a** Spirits sales (per capita 15+) in Luxembourg 2015–2020 divided into three different periods. Index month January = 100. **b** Spirits sales (in litres 100% alcohol per capita 15+) by month in Luxembourg 2018, 2019 and 2020 (until October)

For Luxembourg, spirits sales showed a steady increase from 2015 to February 2019. In March–May 2020 Luxembourg experienced a dramatic decline in spirits sales with a rebound during the summer months of June, July and August. Looking at the full January–October period, spirit sales remain on the same level as 2019, representing a notable break in the trend of increasing spirit sales (Fig. 3a-b). This change of trend is further confirmed in the SARIMA analysis in Table 2.

**Table 2:** Estimated effects of the pandemic (March–December 2020) on the RAC, total and beverage specific. Interrupted time series analyses ARIMA-SARIMA^a,b^

	EST^b^	SE	*p*	Estimated effects in %
**BE**				
Total	−0.108	0.036	**0.003**	−10.2
Beer	−0.230	0.045	**< 0.001**	−20.5
Spirits	0.041	0.034	0.221	4.2
Wine	−0.033	0.029	0.260	2.9
**DE**				
Total	−0.049	0.011	**< 0.001**	−4.8
Beer	−0.060	0.012	**< 0.001**	−5.8
Spirits	−0.018	0.016	0.249	5.1
**DK**				
Total	0.012	0.037	0.741	1.2
Beer	−0.047	0.048	0.333	−4.6
Spirits	−0.041	0.036	0.255	−4.9
Wine	0.081	0.059	0.169	8.4
**EE**^3^				
Total	−0.034	0.224	0.880	−3.3
Beer	−0.223	0.351	0.524	−20.0
Spirits	0.048	0.235	0.838	−8.8
Wine	−0.072	0.097	0.459	−6.9
**FI**				
Total	0.022	0.010	**0.011**	2.2
Beer	0.021	0.020	0.294	2.1
Spirits	0.020	0.029	0.483	2.0
Wine	0.041	0.019	**0.027**	4.0
**FR**				
Total	−0.070	0.018	**< 0.001**	−6.8
Beer	−0.093	0.036	**0.009**	−8.9
Spirits	−0.014	0.022	0.531	−1.4
Wine	−0.077	0.022	**< 0.001**	− 7.4
**IE**				
Total	−0.081	0.019	**< 0.001**	−7.8
Beer	−0.234	0.031	**< 0.001**	−20.9
Spirits	−0.085	0.034	**0.013**	−6.4
Wine	0.105	0.031	**< 0.001**	11.1
**LT**^c^				
Total	−0.138	0.162	0.392	−12.9
Beer	0.037	0.078	0.632	3.8
Spirits	−0.306	0.197	0.121	−26.4
Wine	0.157	0.297	0.596	17.0
**LV**^c^				
Total	−0.167	0.032	**< 0.001**	−14.2
Beer	−0.183	0.065	**0.005**	−16.7
Spirits	−0.159	0.053	**0.003**	−14.7
Wine	−0.111	0.043	**0.011**	−10.5
**LU**				
Spirits	−0.193	0.082	**0.019**	−17.6
**NL**^d^				
Total	−0.040	0.037	0.236	−3.9
Beer	−0.091	0.036	**0.013**	−8.7
Spirits	−0.034	0.027	0.220	2.7
Wine	0.017	0.033	0.608	1.7
**NO**				
Total	0.205	0.019	**< 0.001**	22.8
Beer	0.077	0.021	**< 0.001**	8.0
Spirits	0.244	0.018	**< 0.001**	27.6
Wine	0.313	0.019	**< 0.001**	36.5
**PL**^c^				
Total	−0.038	0.019	**0.047**	−3.7
Beer	−0.044	0.030	0.146	−4.3
Spirits	−0.037	0.048	0.447	−3.6
Wine	−0.031	0.065	0.636	−3.1
**SE**				
Total	0.049	0.011	**< 0.001**	5.0
Beer	0.011	0.021	0.586	1.1
Spirits	0.128	0.020	**< 0.001**	13.7
Wine	0.046	0.009	**< 0.001**	4.7

^a^ARIMA-SARIMA analyses in STATA version 13. For model specifications and diagnostic tests, see Additional file 1:Table S2

^b^Estimated impact for March–December 2020 (dummy coded 1, else 0)

^c^Several recent changes in excise duty levels, particularly for EE and LT, complicates the specification of satisfactory SARIMA- models, see specific country analyses below. Final models for EE, LV and LT include data on excise duty levels: beverage specific for beer, spirits and wine and a weighted level in relation to each of these beverages share of the total RAC

^d^Quarterly data 2015.1–2020.4

### Assessment of country specific pandemic impacts

Table 2 shows the estimated effects of the pandemic (March–December 2020) for each main beverage category and total RAC per country (see also Additional file 1: Table S2). In general, the pattern aligns with expectations. For most inflow countries, the impact estimates of the pandemic indicate an increase in RAC. Norway shows, by far, the strongest positive impact estimate on total RAC during the pandemic period (+ 23%) and with significant impacts for all three beverages. Sweden and Finland also show significant pandemic impacts on total RAC but not Denmark and Estonia. For Sweden, both the spirits and wine estimates are significant (+ 14% and + 5%) and for Finland wine estimates are significant (+ 4%). Denmark, as a predicted inflow country, does not show any significant effects.

The two typical outflow countries – Latvia and Luxembourg - show the strongest negative pandemic impact in decreases of RAC. For the net stable countries, the impact estimates generally indicate a significant negative pandemic impact (decrease) on the total RAC (BE, DE, FR, IE, PL) and the strongest for beer (BE, DE, FR, IE, NL). Ireland and Belgium, with the largest share of on-trade beer sales in 2019 of all 14 countries [26], show particularly strong pandemic impacts on beer (− 20% and − 21% respectively). The impact estimates for wine and spirits are generally non-significant with the exception of Ireland, where the impact estimates show an increase in wine during the pandemic period (+ 11%) and a decrease in spirits (− 6%) and the potential wine outflow country France where the estimates indicate a − 7% decrease for wine during the pandemic period.

Estonia has seen several increases and one decrease in excise duty rates in the past years. Although tax effects are controlled for, spikes in excise duty revenues stemming from wholesalers stocking up or delaying purchases to right before/after larger tax changes complicate the time-series analysis (also the reason why Jan-Feb is not shown for EE, LT and PL in Fig. 2). A detailed inspection of Estonian RAC trends reveal an increase for January–February 2019–2020 for all beverages that is then erased for the period of March–June. August–December 2020 with the same (lower) excise duty levels as for 2019 show lower levels in 2020 than 2019 for all beverages. Taken together, the data points towards Estonia seeing a decrease in RAC as a result of the pandemic for all beverages, but the least for spirits.

Lithuania also saw major excise duty changes in recent years. Monthly data shows a large surge of purchases preceding the taxation increases in 2017 and 2019 and a subsequent lower level of purchasing as stocks are used up. Even controlling for these administrative tax effects, results are insignificant. In addition, looking at 2020 in isolation, or compared to years without tax changes, there is no clearly discernible impact of the pandemic on the overall trend of total RAC.

Excise duty rates were also recently increased in Poland (January 2020) for beer, spirits and wine but the effect on revenue data is smaller than for the two aforementioned Baltic states and with no significant impact on either beverage specific or total RAC. The pandemic impact on total RAC for Poland is estimated at − 4%, but the beverage specific estimates are non-significant.

### Case studies

#### The case of Schleswig-Holstein in Germany

Despite recording significant outflow of alcohol to Scandinavian states, Germany is classified as a stable country in terms of cross-border purchasing. Data on beer sales from German länder provides an opportunity to look at cross-border outflow on a regional level in order to verify this.

The northern German region of Schleswig-Holstein (S-H) is the closest region to Denmark and Sweden. It does, in its northernmost part, contain several hubs for cross-border trade of alcohol concentrated at border crossings [9]. Using monthly data for beer RAC in all German länder 2019 and 2020, S-H can be compared with the rest of Germany. (In the declaration of excise revenues for beer, S-H is reported together with Hamburg - hereafter S-H&H).

**Fig. 4:**
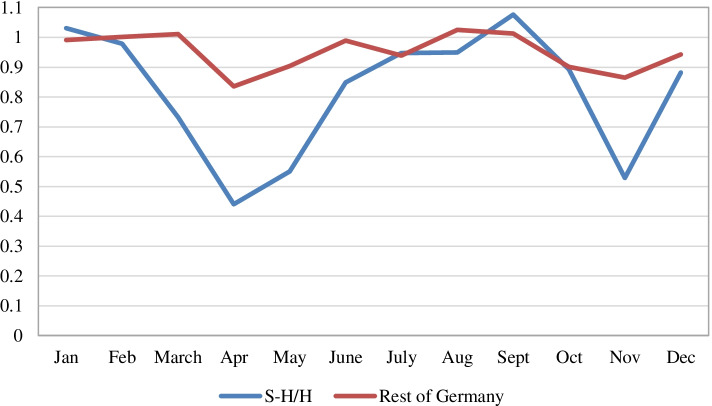
Ratio 2020/2019 per month in beer RAC (revenues) for S-H&H and rest of Germany

As shown in Fig. 4, S-H&H experienced a significantly stronger drop in beer RAC than the rest of Germany, with especially low levels in March–May and November 2020. This is consistent with the characteristics of an outflow country. During the pandemic S-H&H saw a drop in beer RAC of 22% compared to the same period in 2019 whereas the rest of Germany, excluding S-H&H, only saw a drop of 5.8%. Overall, the additional drop in RAC in S-H&H represents less than 1% of the total German beer revenues supporting the classification of Germany as a country with low net cross-border flows.

#### Reallocation of purchases - the case of Sweden

The observed increases in RAC for inflow countries during the pandemic is testament to significant reallocation of alcohol purchases taking place as a result of closed borders. It is, however, difficult to draw any conclusions with regard to total consumption purely from sales data. Any effect on total consumption depends on the substitution ratio between cross-border and domestic purchases, i.e. the ratio of change in domestic sales volumes for every change in cross-border purchase volumes by its residents.

The national Swedish monitor study notes a drop in total alcohol consumption of 6% from 2019 to 2020 [27], stronger than the identified decrease in sales in comparable stable countries. In Finland, another inflow country, the national survey on total alcohol consumption notes a decrease of 5.2% [28], also suggesting that the drop in cross-border purchases of 52% in 2020 [29] was only partially replaced by domestically purchased alcohol.

Using Sweden as a case-study, rough estimates of substitution effects can be obtained by combining the  data on RAC with (a) data on volumes of cross-border inflow and outflow from Sweden in the Swedish Monitor study 2019–2020 [27, 30] (b) assessments of the amount of alcohol consumed abroad by Swedish residents and by visitors in Sweden and (c) predicted changes in RAC based on countries with low net levels of cross-border shopping that are comparable to Sweden in terms of on-trade share for beer [26] as well as pandemic restrictions [3]. These countries, in this case data from Germany and Poland,^4^ can then be used as ‘control sites’ predicting the change in domestic sales in Sweden due to those pandemic restrictions not related to cross-border shopping.

As is shown in Table 3, the substitution ratios indicate an additionality in cross-border purchasing. This applies for total purchases as well as for individual beverages apart from wine, likely explained by some substitution from other beverage categories to wine. Using the same ‘control site’ methodology, it is also possible to estimate the excise duty effect of the reallocated purchases, with Sweden showing a significant increase (+ 7.6%) in excise duty revenue in 2020.

**Table 3:** Changes in cross-border inflow, consumption abroad, domestic sales (by Swedish residents) and estimated substitution ratios and changes in revenues 2020/2019. (Substitution ratios (D) below a 100% indicate that cross-border sales are only partly replaced by domestic purchases and vice versa)

Changes 2019–2020 (in million litres 100% alc.) and estimated substitution rates	Beer	Spirits	Wine	Total
(A) Change in cross-border inflow^**a**^ and tourist consumption abroad^**b**^	−4.0	−3.8	−3.2	−11.0
(B) Change in domestic sales to Swedish residents^**c**^	+ 2.5	+ 1.8	+ 3.7	+ 8.4
(C) Predicted change in domestic sales based on pooled change in DE and PL^**d**^	−1.0	+ 0.03	0.0	−1.4
(D) Substitution ratios^**e**^	85.8%	45.5%	117.6%	89.0%
(E) Actual revenues (change in millions SEK and in %)	+ 216 (+ 5.1%)	+ 556 (+ 12.2%)	+ 362 (+ 6.1%)	1134 (+ 7.6%)
(F) Estimated revenues (change in millions SEK and in %) based on predicted changes in (C)^**d**^	+ 430 (+ 10.6%)	+ 538 (+ 11.8%)	+ 422 (+ 6.1%)	+ 1390 (8.8%)

^**a**^Inflow: data from the Monitor study [27]

^**b**^Using the middle-range alternative estimated by the Monitor project assuming a 25% higher daily consumption abroad than at home [27]. Change 2019–2020: assuming the same percentage decrease in consumption abroad as for total number of trips nights abroad by Swedes (− 84%, [31])

^**c**^RAC minus estimates of Norwegians’ cross-border purchases in SE [30] and other non-Swedish residents’ purchases volumes in Sweden. The latter assumed to be half the volumes of the Swedish consumption volumes abroad, based on the number of tourist days/nights by Swedish tourists abroad and by foreign tourists in Sweden showing a ratio of approx. 2:1. ( [27] 2015 [32]; 2021). Reasonable deviations from this assumption does not more than marginally impact the main results shown in the table

^d^DE and PL 2019–2020: −5% for beer, + 0.4% for spirits, − 1% for wine

^e^Obtained by dividing the difference between observed and predicted sales (B-C) with the change in inflow (A)

## Discussion

The result shows an overall reduction in alcohol sales with 3.6% during the pandemic. Nevertheless, the results seem to heavily differ between different countries, as well as between different types of beverages.

Overall, the results confirm the hypothesised effect of the pandemic on RAC: it fell in countries with expected low levels of cross-border trade and fell strongly in known outflow countries and in a known local outflow region. At the same time, known inflow countries experienced a strong increase in RAC or, in the case for beer, stable levels where other countries saw large sales drops.

The results also align rather well when comparing individual beverages and countries. Beer RAC decreased the most, which, most likely, can be attributed to on-trade restrictions impacting beer more strongly than other beverages. The conclusion is further strengthened by the stronger-than-average beer decrease for Belgium and Ireland as the countries with the strongest on-trade beer share pre-pandemic among the countries studied [26]. The dual effect of restrictions on beer serve to explain the stability of beer sales in inflow countries, as increases from cross-border reallocations are counterbalanced by decreases from on-trade restrictions. This means that even stable levels of beer sales, such as in Denmark, can be indicative of cross-border reallocations. Overall, there is strong evidence that on-trade restrictions affected beer sales more than other beverage categories and contributed to the overall decrease in RAC. This conclusion holds up even when taking into account smaller substitution effects between beverage categories.

Looking at the case of Sweden, the study supports previous scientific findings of a sizable additionality in cross-border purchasing with substitution ratios below 100 and positive effects on excise duty revenue. To our knowledge, this is the first time such effects can also be confirmed for restrictions to cross-border purchasing, which has important implications for policy-making. These are conservative estimates as they are modelled on a full calendar year in 2020, i.e. including the pre-pandemic months of January and February.

On an individual country level perspective, some noticeable cases can be seen as indicative of cross-border trade flows in need of further research: e.g. the increase in wine RAC in Ireland. Although likely, in part, representative of a switch from on-trade beer to off-trade wine sales, the trend does resemble that of an inflow country (see also Additional file 1: Table S1).

For Norway the large increases in total and beverage specific RAC during the pandemic stand out compared to other inflow countries. This is likely an effect of Norway being at the top of the Nordic-Baltic cross-border purchasing chain with little to no outflow of alcohol. It can also be explained by Norway having larger price differentials for alcohol and other products to neighbouring Sweden and Denmark than any intra-EU neighbours [13, 33] despite price differentials being somewhat counterbalanced by more restrictive rules for transporting alcohol across borders. As official estimates of cross-border inflow volumes are significantly lower than the observed RAC increases highlights the need to revisit official cross-border estimates for Norway.

France may be another outlier when it comes to wine, experiencing a strong decrease in wine RAC where most other countries show largely unchanged sales trends. This can likely be attributed to wine-oriented drinking patterns being stronger in France as well as the country experiencing higher wine RAC from tourists compared to other countries in the study.

Latvia, as an outflow country, shows strong negative impact estimates, but these are harder to identify in ocular inspections between 2019 and 2020. Seen from a longer time period, nonetheless, breaks in the time series do appear. One possible explanation to this is the reduction of excise duty rates in Estonia in July 2019, which reduced cross-border shopping from Latvia to Estonia. While not a topic for this study, future studies on how tax changes in one country affect sales in a neighbouring country would be of interest in order to better understand changes in cross-border alcohol sales.

### Limitations

The study does classify all months from March to December 2020 as belonging to the pandemic period. The degree of imposed restrictions, including border closures, did however differ during this period with the summer months (May–August) representing a less restrictive period for most countries without much variation [3]. As such this study uses the same intervention period and the same degree of exposure for all 10 months across all the countries. A more sensitive and country-specific intervention measure would most likely show stronger pandemic effects, as the less restrictive summer months act as counterweights in the current measure. However, such a measure would complicate comparisons between countries.

The study also does not consider the efforts made by countries or companies to increase availability and/or reduce the price of alcohol to help businesses cope with the pandemic restrictions. This may have had minor effects on consumption in some countries, but it is unlikely to affect the overall results and conclusions.

Although possible to capture as part of cross-border outflow in the methodology, tourism-related effects on consumption in a country is not the focus of the paper. For most countries in the study, changes to tourism flows will only have a minor impact on consumption. However, for some countries, such as France, with a sizable tourism sector in general, and wine-related tourism sector in particular [34–36] this effect is likely stronger.

### Policy implications

The results strengthen the case for current cross-border alcohol taxation rules in the EU having negative public health and tax revenue effects as well as creating market distortions. To address these effects, the results lend support to the idea of restricting cross-border purchasing rules on a national and EU-level. Nationally, it could take the form of restrictive national interpretations of EU-rules of when alcohol can be brought into a country without further payment of excise duty, as well as investment in enforcement capability and sanctions to deter abuse of cross-border excise differentials.

On an EU-level, the results bring attention to the need for a review of EU rules for cross-border alcohol purchases. Although the effect of such reforms would be smaller than what has been observed during the pandemic, there is reason to believe such a restriction in the economic availability of cross-border alcohol purchasing would reallocate excise revenue and strengthen the effect of national pricing policies as instruments to reduce alcohol-related harm. Over time, such a reform could provide lasting relief for national healthcare systems and have an impact on alcohol-related mortality, primarily in high-excise duty EU Member States.

## Supplementary Information

**Additional file 1.**

## Data Availability

The datasets generated and analysed during the study are not publicly available in order to avoid misuse by economic operators in the alcohol sector but are available from the corresponding author on reasonable request.

## Footnotes

1 This does not apply to Norway, but the variable on alcohol price differences to neighbouring countries is significantly stronger [13] and further reinforced by the price differences for other goods increasing the pull-factor for cross-border shopping in general [14].

2 Total per capita alcohol consumption is here defined as the total amount of alcohol consumed per person (15+) irrespective of the source of acquisition (see also the WHO definition [15])

3 All volume references will hereby refer to litres 100% alcohol per capita 15 years or older

4 The identified additionality is also robust to the inclusion of other sets of comparable stable countries.
