# Supplementary material for: Alcohol consumption and closed borders - how COVID-19 restrictions have impacted alcohol sales and consumption in Europe
Source: BMC Public Health. 2022 Apr 8;22:692. doi: 10.1186/s12889-022-13014-1 (PMC8993209; doi:10.1186/s12889-022-13014-1)
Supplement: Supplementary file 1 — Additional file 1. [file 12889_2022_13014_MOESM1_ESM.docx]

**Additional file 1**

**Table A1.** Preliminary categorisation of countries in relation to cross-border purchase flows.

| **Study countries** | **Category** | **Comments** |
| --- | --- | --- |
| Belgium (BE)* | Net stable/  inflow | Small inflow from LU of spirits and sparkling wine (1), |
| Germany (DE) * | Net stable / outflow | Outflow from northern DE to Nordic countries based on the presence of large border shops and the presence of a system of packaging fee (Pfand) waiver for Scandinavian customers (2). Outflow is likely small compared to total sales. |
| Denmark (DK) | Inflow | Inflow from DE. Some outflow to SE and NO. Largest cross-border purchasing share for beer followed by spirits. Cross-border inflow estimated at 19% of total consumption in 2016 (3). |
| Estonia (EE) | Inflow/  outflow | Inflow from LV, outflow to FI. Net inflow of beer with net outflow of spirits. In total: more outflow than inflow 2019 and 2020 (4). |
| Finland (FI) | Inflow | Inflow from EE (50% of total inflow) followed by LV, SE and the Åland Islands. Largest cross-border purchasing share for spirits followed by beer, wine, long drinks, cider and intermediate products (5). Cross-border inflow was reduced by 52% in 2020 and the share of unrecorded alcohol consumption dropped from 18% to 12% of total consumption (6, 7, 8). |
| France (FR)* | Net stable / outflow | Outflow to UK and IR confirmed by newspaper reports and presence of large border shops at ferry crossings. Some inflow expected from LU & DE due to price differences. Outflow patterns are also expected due to high tourist presence in FR (9). |
| Ireland (IE)* | Net stable / Inflow | Small inflow from Northern Ireland subject to currency fluctuations (10). Likely inflow of wine from FR based on presence of large shops at popular ferry crossings. |
| Lithuania (LT)* | Net stable / inflow | Reports of inflow from LV and PL (11, 12). Evidence of border shops in LV targeting LT customers. |
| Latvia (LV)* | Outflow | Outflow to primarily to EE and some to FI and LT. (See also FI, LT, EE) |
| Luxembourg (LU)* | Outflow | Likely large outflow of all beverages, but notably wine, sparkling wine and spirits based on presence of large border shops. The share of cross-border purchases recognised by the WHO as a reason for difficulties of estimating true levels of domestic consumption (13). |
| The Nether-lands (NL)* | Net stable | Low net levels of cross-border alcohol purchases found (14) |
| Norway (NO) | Inflow | Inflow primarily across the border from SE and duty-free purchases at airports. In 2018, duty-free purchases of alcohol at airports was estimated at 0.38 litres and cross-border inflow from SE was at 0.62 litres (per capita 15+) (15) |
| Poland (PL)* | Net stable | Outflow to LT and DE. Likely small in comparison to total sales in the country |
| Sweden (SE) | Inflow | Inflow from mainly DK, FI, DE, PL as well as the Åland Islands (FI) but largest share from DE. Outflow to NO: a fifth of the size of total inflow. Largest cross-border purchasing share for spirits, followed by beer and wine. Cross-border inflow, including smuggling) estimated at 17% of total consumption in 2019 and 8% in 2020. Total RAC increased by 5% and total alcohol consumption dropped by 6% during 2020 (16). |

* inferred from triangulation. Where evidence of inflow is found, this is also taken as evidence of the presence of outflow. Triangulated sources can be found either in outflow or inflow country comments.

**Table A2**. Data on alcohol excise duty revenues per country^1^

| **Country** | **Population in million 15+ (2020)** ^2^ | **Source data** | **Comments** | |
| --- | --- | --- | --- | --- |
| BE | 9.58 | Beer, still wine and other fermented beverages, sparkling wine and other sparkling fermented beverages, ethyl alcohol (income)  Conversion factor for wine: 12.5% | Beer taxed per degree plato*  Represents month of declaration in national accounts (t) | |
| DE | 71.78 | Beer (volume finished product), ethyl alcohol (income)  Wine (volume finished product) Conversion factor for wine: 11%^3^ | Beer volume converted to pure alcohol using an average beer strength of 5.2 ABV  Periodisation issue for spirits December 2020, average December-20/January-21 used.  Data adjustment unknown.  Wine data obtained only as yearly sales estimates as Germany does not tax wine. | |
| DK | 4.87 | Beer, wine, ethyl alcohol (income)  Conversion factor for wine: 12.5% | Represents month of declaration in national accounts (t).  December values 2018/19/20 contain January the following year. | |
| EE | 1.11 | Beer, wine, ethyl alcohol, intermediate and fermented products (income)  Conversion factor for wine: 12.2% | Represents month of declaration in national accounts (t) | |
| FI | 4.65 | Beer, wine, fermented beverages, ethyl alcohol (alcohol volume), intermediate products  Conversion factor for wine: different for different categories, main category: 12.7% | Represents month of declaration in national accounts (t) | |
| FR |  | Ethyl alcohol, wine (including other fermented beverages), beer, intermediate products (income)  Conversion factor for wine: 12.8% |  | |
| IE | 3.96 | Beer, wine, ethyl alcohol, cider (income)  Conversion factor for wine: 12.5 | Represents month of declaration in national accounts (t) | |
| LT | 2.37 | Beer, wine, intermediate products, ethyl alcohol (income)  Conversion factor for wine: 10.0% | Represents month of release for consumption (t-1).  Very strong spikes in excise duty income month before excise duty increases. | |
| LU | 0.53 | Ethyl alcohol (alcohol volume) | Monthly data 2015-2020, November/December 2020 missing. Data time-adjustment unknown. | |
| LV | 1.60 | Beer, all other alcohol products (income)  Conversion factor for wine: 12.3% | Non-beer beverages declared together. Ratio on spirits/wine based on annual data ((17) 2015-2018, (18) 2019-2020) applied on monthly data  Data time-adjustment unknown. | |
| NL | 14.68 | Beer, Still wine, Sparkling wine, Ethyl alcohol  Conversion factor for wine: 12.5% | Beer taxed per degree plato* Quarterly data from Statistics Netherlands database Statline-CBS. This data is based on excise duty payments. Monthly data was also available from the Directorate-General for Tax and Customs Administration of the Ministry of Finance. This data is based on volumes obtained from tax declarations.  The two datasets do not match each other, with a particularly large gap for beer. The quarterly data exhibits a strong decrease in beer sales during the pandemic whereas the monthly data shows a slight increase. As quarterly data is based on actual payments, it can be affected by the Dutch excise duty payment holiday introduced as part of the support package for the Horeca-sector during the pandemic, meaning it would underestimate sales. The decrease in sales is nonetheless confirmed by reports from Dutch Brewers and beer import statistics from CBS-statline. As no external datapoints can explain the lack of decrease in the monthly data, we chose to include the quarterly data in the analysis, despite the risk of underestimating beer sales.  Monthly data represents month of declaration in national accounts (t), quarterly data represents quarter of registration of payment in national accounts. | |
| NO | 4.44 | Beer, wine/ethyl alcohol together (income)  Conversion factor for wine: already in pure alc | Represents month of declaration in national accounts (t)  Quarterly data on ratio spirits/wine applied to separate monthly data. | |
| PL | 32.10 | Beer, wine (including intermediate products and other fermented beverages), Ethyl alcohol (income)  Conversion factor for wine: 12.0% | Beer taxed per degree plato*  Data adjustment unknown. | |
|  |  |  |  | |
| SE | 8.49 | Beer, wine, ethyl alcohol, intermediate products (income) Conversion factor for wine: 12.8% | Represents month of declaration in national accounts (t) | |
|  |  |  |  | |
| ^1^ All data based on alcohol excise duty receipts (tax records 2015-2020). The tax records model of estimating yearly alcohol consumption data is widely used in countries where alcoholic beverages are taxed. Albeit with a slightly larger margin of error, it can also be applied on monthly data. Alcohol excise duty is levied when the products are released for consumption (19). In practice, this means that excise duty is levied before products are sold to the consumer. However, as changes in alcohol consumption would feedback into changes in purchases and supplier orders, alcohol excise duty data is still an accurate proxy of sales changes also on a monthly basis.  For wine, the conversion from litres finished product sold to pure alcohol is done according to national average strength conversion factors as reported to the OECD (20) or national reports and studies. Where no such reporting exists the conversion factor of 12.5% has been used. For wine Germany, see Kuitunen-Paul *et al.* (21)  ^2^ Population aged 15 and over in millions, year 2020 (22)  *A conversion factor of 2,5 is used to convert excise duty based on degree plato to degree alcohol | | | |  |

**Table A3.** Model specifications for each country and beverage Interrupted time series analyses (ARIMA-SARIMA)

|  | Model specifications and comments | Box-Ljung, Q, p-value^1^ |
| --- | --- | --- |
| **BE** | All from 2016 because of big change in taxation November-15. |  |
| Total | arima(1,0,0) sarima(0,1,1,12), | 2.271, 0.132 |
| Beer | arima(0,0,0) sarima(1,1,1,12 | 1.524, 0.217 |
| Spirits | arima(0,0,0) sarima(1,1,1,12 | 0.242, 0.623 |
| Wine | arima(1,0,0) sarima(0,1,1,12) from 2016 | 3.654; 0.056 |
| **DE** |  |  |
| Total | Beer and spirits summed arima(1,0,0) sarima(0,1,1,12) | 0.074; 0.786 |
| Beer | arima(2,0,0) sarima(0,1,1,12) | 0.034; 0.854 |
| Spirits | arima(1,0,0) sarima(0,1,0,12) | 1.639; 0.200 |
| **DK** |  |  |
| Total | arima(1,0,0) sarima(0,1,1,12) | 0.002, 0.962 |
| Beer | arima(1,0,0) sarima(0,1,1,12). | 0.003, 0.966 |
| Spirits | arima(1,0,0) sarima(0,1,1,12) | 0.003, 0.958 |
| Wine | arima(2,0,0) sarima(0,1,1,12) | 0.004; 0.951 |
| **EE** |  |  |
| Total | arima(1,0,0) sarima(1,1,0,12) Overall alcohol taxation data included as a weighted sum of beer, spirits and wine taxation levels in relation to their RAC in relation to total RAC | 0 .036, 0.849 |
| Beer | arima(1,0,0) sarima(0,1,1,12). Beer taxation data included | 0 .288; 0.591 |
| Spirits | arima(0,0,1) sarima(1,1,0,12). Spirits taxation data included | 0.001; 0.970 |
| Wine | arima(1,0,0) sarima(0,1,1,12) Wine taxation data included | 0 .041, 0.839 |
| **FI** |  |  |
| Total | arima(1,0,0) sarima(0,1,1,12), | 1.048, 0.306 |
| Beer | arima(1,0,0) sarima(0,1,1,12 | 0.062, 0.804 |
| Spirits | arima(2,0,0) sarima(1,1,0,12) | 0.002, 0.966 |
| Wine | arima(1,0,0) sarima(0,1,1,12). Wine taxation data included | 0.063, 0.802 |
| **FR** |  |  |
| Total | arima(2,0,0) sarima(0,1,1,12) | 0.001; 0.972 |
| Beer | arima(1,0,0) sarima(0,1,1,12), | 0.006, 0.937 |
| Spirits | arima(2,0,0) sarima(0,1,1,12) | 0.028, 0.868 |
| Wine | arima(2,0,0) sarima(0,1,1,12) | 0.000, 0.997 |
| **IE** |  |  |
| Total | arima(1,0,1) sarima(0,1,0,12) | 0.061; 0.805 |
| Beer | arima(2,0,0) sarima(0,1,1,12) | 5.427; 0.020 |
| Spirits | arima(1,0,0) sarima(0,1,1,12) | 2.587; 0.108 |
| Wine | arima(1,0,0) sarima(0,1,1,12) | 0.022; 0.882 |
| **LT** |  | 0.008, 0.927 |
| Total | arima(1,0,0) sarima(1,1,0,12). Overall alcohol taxation data included as a weighted sum of beer, spirits and wine taxation levels in relation to each beverage share of total RAC. Dummy for big increase in beer excise duties in Feby-2017 and Feby-2019, one month before beer tax changes in March 2017, 2019. | 0.066, 0.798 |
| Beer | arima(2,0,0) sarima(1,1,0,12), Beer taxation data included and a dummy for big increase in beer excise duties in February-2017 one month before beer tax change in March 2017. | 0.017, 0.896 |
| Spirits | arima(1,0,0) sarima(1,1,0,12), Dummy for big increase in beer excise duties in February-2017 and February-2019 one month before beer tax changes in March 2017, 2019. | 0.003, 0.954 |
| Wine | arima(2,0,0) sarima(1,1,0,12), Dummy for big increase in wine excise duties in February-2017) one month before wine tax change in March 2017. | 0.000, 0.989 |
| **LV** |  |  |
| Total | arima(1,0,0) sarima(0,1,1,12). Overall alcohol taxation data included as a weighted sum of beer, spirits and wine taxation levels in relation to each beverage share of total RAC. | 0.004, 0.951 |
| Beer | arima(2,0,0) sarima(0,1,1,12). Beer taxation data included | 0.041, 0.839 |
| Spirits | arima (1,0,0) sarima(0,1,1,12) Spirits taxation data included | 0.008, 0.932 |
| Wine | arima(2,0,0) sarima(0,1,1,12) Wine taxation data included | 0.002, 0.978 |
| **LU** | Impact period: March-October 2020 |  |
| Spirits | arima(1,0,0) sarima(0,1,1,12) | 0.798; 0.372 |
| **NL** | Impact period: Q2-Q4 (April-December) 2020 |  |
| Total | arima(2,0,0) sarima(1,1,0,4) | 0.027, 0.870 |
| Beer | arima(0,0,0) sarima(0,1,0,4) | 0.023, 0.880 |
| Spirits | arima(2,0,0) sarima(0,1,1,4) | 0.003, 0.960 |
| Wine | arima(1,0,0) sarima(1,1,0,4)) | 0.023, 0.880 |
| **NO** |  |  |
| Total | arima(2,0,0) sarima(0,1,1,12) | 0 .301, 0.583 |
| Beer | arima(2,0,0) sarima(0,1,1,12) | 0 .161; 0.689 |
| Spirits | arima(2,0,0) sarima(0,1,1,12) | 0.340; 0.560 |
| Wine | arima(2,0,0) sarima(0,1,1,12) | 0.322, 0.570 |
| **PL** |  |  |
| Total | arima(1,0,0) sarima(0,1,1,12) | 0.870; 0.351 |
| Beer | arima(2,0,0) sarima(0,1,1,12) | 0.302, 0.582 |
| Spirits | arima(1,0,0) sarima(0,1,1,12) | 4.451; 0.035 |
| Wine | arima(2,0,0) sarima(0,1,1,12) | 0.899, 0.343 |
| **SE** |  |  |
| Total | arima(2,0,0) sarima(0,1,1,12) | 0.486, 0.486 |
| Beer | arima(2,0,0) sarima(0,1,1,12) | 1.907; 0.167 |
| Spirits | arima (1,0,0) sarima(0,1,1,12) | 0.452; 0.502 |
| Wine | arima(2,0,0) sarima(0,1,1,12) | 3.123; 0.077 |
|  |  |  |

^1^In ARIMA-modelling, the error term (noise structure), which includes explanatory variables not included in the model, is allowed to have a temporal structural that is modelled and estimated in terms of autoregressive and/or moving average parameters. This increases the reliability of the model estimates. An important criterion of model fit is that the residuals are white noise. This was determined by means of the Box-Ljung Q statistics.

**References**

1. Hindriks J, Serse. Heterogeneity in the tax pass-through to spirit retail prices: Evidence from Belgium. Journal of Public Economics, 2019;176;142–160;doi: 10.1016/j.jpubeco.2019.06.009.
2. European Court of Justice. Judgement of 9 June 2021. 2021. Dansk Erhverv, T‑47/19 ECLI:EU:T:2021:331.
3. Skatteministeriet (Denmark). (2017). Bilag – Status over grænsehandel 2017. 2017. <https://www.skm.dk/media/6411/bilag-status-over-graensehandel-2017.pdf>. Accessed 12 aug 2021.
4. Eesti Konjunktuuriinstituut. Eesti alkoholiturg, alkoholi tarbimine ja alkoholipoliitika 2020. aastal *[Estonian alcohol market, alcohol consumption and alcohol policy in 2020]*. 2021. <https://www.sm.ee/sites/default/files/news-related-files/eesti_alkoholiturg_alkoholi_tarbimine_ja_alkoholipoliitika_2020_uus.pdf>. Accessed 18 aug 2021.
5. RP 145/2020 rd. Regeringens proposition till riksdagen med förslag till lag om ändring av bilagan till lagen om accis på alkohol och alkoholdrycker. [The proposal of the government to the Finnish parliament with a suggestion to revise the appendix of the law on taxes on alcohol and alcoholic beverages]. Parliament of Finland. 2020. <https://finlex.fi/sv/esitykset/he/2020/20200145> Accessed 12 aug 2021.
6. THL. Alkoholijuomien kulutus 2019 Alkoholin kokonaiskulutus väheni 3,9 prosenttia *[Consumption of alcoholic beverages in 2019. Total alcohol consumption decreased by 3.9 per cent]*. 2020. <https://www.julkari.fi/bitstream/handle/10024/139553/Tr06_2010_Alkoholijuomien%20kulutus%202019.pdf?sequence=1&isAllowed=y>. Accessed 12 aug 2021.
7. THL. Alkoholijuomien kulutus 2020. Alkoholin kokonaiskulutus väheni 5,2 prosenttia. *[Consumption of alcoholic beverages in 2020.Total alcohol consumption decreased by 5.2 percent]*. 2021. <https://www.julkari.fi/bitstream/handle/10024/141145/tr7_21.pdf?sequence=1&isAllowed=y>. Accessed 12 aug 2021
8. THL. Alkoholijuomien matkustajatuonti 2020. Alkoholin matkustajatuonti puolittui vuonna 2020*. [Passenger imports of alcoholic beverages by 2020. Passenger imports of alcohol halved in 2020]*. 2021. <https://www.julkari.fi/bitstream/handle/10024/141020/Alkoholijuomien%20matkustajatuonti%202020.pdf?sequence=1&isAllowed=y>. Accessed 12 aug 2021.
9. La Presse de la Manche. Britanniques et Irlandais en sont friands à Cherbourg: les vins français à l’épreuve du Brexit *[Brits and Irish have an appetite for Cherbourg: French wines face the test of Brexit]*. La Presse de La Manche. 2019. October 31.
10. McNeilly C. *Border towns boom time as southern shoppers flock to Northern Ireland*. Belfast Telegraph. 2016. <https://www.belfasttelegraph.co.uk/business/news/border-towns-boom-time-as-southern-shoppers-flock-to-northern-ireland-35192961.html>. Accessed 12 aug 2021.
11. Carlsberg, Regioplan Policy Research, EY. Economic effects of high excise duties on beer. Amsterdam. 2018. <https://www.regioplan.nl/wp-content/uploads/data/file/2017/17155-Final-Report-Economic-effects-of-high-excise-duties-on-beer-in-the-Baltic-States-Regioplan.pdf>. Accessed 12 aug 2021.
12. Drug Tobacco and Alcohol Department Lithuania (NTAKD). Cross-border alcohol purchases in the Baltic countries. 2019. Event.
13. OECD, European Union. Health at a Glance: Europe 2018 State of Health in the EU cycle. Paris: OECD Publishing. 2018; doi: 10.1787/23056088.
14. Institute for Transnational and Euregional cross border cooperation and Mobility (Maastricht University). *Cross-Border Impact Assessment 2018*. Maastricht University. 2018. <https://www.maastrichtuniversity.nl/research/item/research/archive/archive-item-cross-border-impact-assessment#cbia2018>. Accessed 12 aug 2021.
15. Guttormsson U, Trolldal B. Norsk gränshandel med alkohol i Sverige [*Norweigan cross border shopping of alcohol in Sweden]*. Rapport 190. 2020. Stockholm. [https://www.can.se/app/uploads/2020/01/can-rapport-190-norsk-granshandel-med-alkohol-i-sverige-1.pdf. Accessed 12 aug 2021](https://www.can.se/app/uploads/2020/01/can-rapport-190-norsk-granshandel-med-alkohol-i-sverige-1.pdf.%20Accessed%2012%20aug%202021).
16. Trolldal B, Åström V. Alkoholkonsumtionen i Sverige 2001–2020. [*Alcohol consumption in Sweden 2001–2020*]. 2021. Rapport 202. Stockholm. <https://www.can.se/app/uploads/2021/09/can-rapport-202-alkoholkonsumtionen-i-sverige-2001-2020.pdf> Accessed 25 nov 2021
17. The Global Health Observatory, World Health Organisation. NCD Risk Factors - Alcohol, recorded per capita (15+) consumption (in litres of pure alcohol). 2021. [Alcohol, recorded per capita (15+) consumption (in litres of pure alcohol) (who.int)](https://www.who.int/data/gho/data/indicators/indicator-details/GHO/alcohol-recorded-per-capita-(15-)-consumption-(in-litres-of-pure-alcohol)). Accessed 26 nov 2021.
18. Valsts ieņēmumu dienests. Akcīzes preču aprites daļa - Alkohola aprite - 1.pielikums *[ Part of the movement of excisable goods - alcohol circulation Annex 1]*. 2019. <https://www.vid.gov.lv/sites/default/files/alkohola_aprite_2019_06.pdf> Accessed 6 sept 2021.
19. Council of the European Union. Council Directive 2008/118/EC of 16 December 2008 concerning the general arrangements for excise duty and repealing Directive 92/12/EEC. *OJ*. Brussel. 2019;(L9);12–30.
20. OECD. OECD Health Statistics 2021 - Definitions, Sources and Methods. 2021. <https://www.oecd.org/els/health-systems/Table-of-Content-Metadata-OECD-Health-Statistics-2021.pdf> Accessed 19 aug 2021.
21. Kuitunen-Paul S. *et al.* Assessment of alcoholic standard drinks using the Munich composite international diagnostic interview (M-CIDI): An evaluation and subsequent revision. International Journal of Methods in Psychiatric Research. 2017;26;Article e1563. <https://onlinelibrary.wiley.com/doi/epdf/10.1002/mpr.1563>.
22. Eurostat Data Browser. Eurostat. Population on 1 January by age group and sex. 2021. <https://ec.europa.eu/eurostat/databrowser/view/DEMO_PJANGROUP__custom_1777403/default/table?lang=en>. Accessed 26 aug 2021.
